# Evaluation of Cell-Specific Alterations in Alzheimer’s Disease and Relevance of In Vitro Models

**DOI:** 10.3390/genes14122187

**Published:** 2023-12-07

**Authors:** Giorgio Guido, Katia Mangano, Lyubka Tancheva, Reni Kalfin, Gian Marco Leone, Andrea Saraceno, Paolo Fagone, Ferdinando Nicoletti, Maria Cristina Petralia

**Affiliations:** 1 Department of Biomedical and Biotechnological Sciences, University of Catania, Via S. Sofia 89, 95123 Catania, Italy; giorgio_guido@outlook.it (G.G.); kmangano@unict.it (K.M.); g.marco-94@outlook.it (G.M.L.); andreasara96@gmail.com (A.S.);; 2 Department of Biological Effects of Natural and Synthetic Substances, Institute of Neurobiology, Bulgarian Academy of Sciences, Acad. Georgi Bonchev Str. Block 23, 1113 Sofia, Bulgaria; lyubkatancheva@gmail.com (L.T.); reni_kalfin@abv.bg (R.K.); 3 Department of Healthcare, South-West University “Neofit Rilski”, Ivan Mihailov Str. 66, 2700 Blagoevgrad, Bulgaria; 4 Department of Clinical and Experimental Medicine, University of Messina, 98122 Messina, Italy

**Keywords:** Alzheimer’s disease, neurodegeneration, in vitro models

## Abstract

Alzheimer’s disease (AD) is a neurodegenerative disorder classically characterized by two neuropathological hallmarks: β-amyloid plaques and tau tangles in the brain. However, the cellular and molecular mechanisms involved in AD are still elusive, which dampens the possibility of finding new and more effective therapeutic interventions. Current in vitro models are limited in modelling the complexity of AD pathogenesis. In this study, we aimed to characterize the AD expression signature upon a meta-analysis of multiple human datasets, including different cell populations from various brain regions, and compare cell-specific alterations in AD patients and in vitro models to highlight the appropriateness and the limitations of the currently available models in recapitulating AD pathology. The meta-analysis showed consistent enrichment of the Rho GTPases signaling pathway among different cell populations and in the models. The accuracy of in vitro models was higher for neurons and lowest for astrocytes. Our study underscores the particularly low fidelity in modelling down-regulated genes across all cell populations. The top enriched pathways arising from meta-analysis of human data differ from the enriched pathways arising from the overlap. We hope that our data will prove useful in indicating a starting point in the development of future, more complex, 3D in vitro models.

## 1. Introduction

Alzheimer’s disease (AD) is a neurodegenerative disorder that primarily affects cognitive function and memory. Hypotheses of AD pathophysiology include amyloid cascade, inflammation, vascular and infection factors [1]. AD is classically characterized by three neuropathological hallmarks: β-amyloid plaques, tau tangles and neuroinflammation [2]. Plaques are extra-cellular and composed of aggregated Aβ. Amyloid can also deposit in vessel walls, causing cerebral amyloid angiopathy, the most common cause of hemorrhagic stroke in the old population [3]. Aβ can vary from 38 to 43 AA, depending on the way it is cut. It originates from an amyloid precursor protein (APP) due to the activity of three enzymes (α, β and γ secretases) that cut it at specific sites. The APP process can follow either the amyloidogenic or the non-amyloidogenic pathway [4]. The latter is mediated by α and γ secretases. Around 90% of the Aβ we produce is in the form of Aβ-40. The form Aβ-42 has been found to be the most toxic and originates from the cutting of the amyloid precursor APP by the β-secretase, and subsequently by the γ-secretase (amyloidogenic pathway). The longer the peptide, the higher the probability that it aggregates [5]. Aβ deposition triggers homeostatic responses like the unfolded protein response (UPR), a stress response that aims to restore protein homeostasis [6] and seems to be an innate immune mechanism to a perceived or real immune challenge. Indeed, Aβ entraps pathogens and elicits neuroinflammation to fight against them [7]. Until the early 2000s, Aβ was the only focus of AD research, but tangles became more and more studied in subsequent years. Tangles contain hyperphosphorylated tau, a microtubule-binding protein, which plays an important role in intracellular transport [8]. Tangles are not uniformly present in the brain. The entorhinal cortex, hippocampus, part of the neocortex and the nucleus basalis of Meynert are affected. The first two underlie memory defects. The involvement of the basal forebrain via cholinergic abnormality contributes to cognitive defects. The cholinergic involvement distinguishes AD from normal aging, related to fronto-striatal involvement. Thus, tangles better correlate with symptoms than Aβ plaques [9]. The tau spreading pattern was thought to be the same in all AD patients and formalized by Braak stages [10]. However, recent data from tau-PET studies show different spatio-temporal trajectories [11]. In particular, a recent study identified four tau deposition trajectories: limbic-predominant, medial temporal-lobe-sparing, posterior and lateral temporal patterns. No dominant pattern was identified, although the limbic pattern was the most frequent. The third hallmark of AD pathogenesis is inflammation. Microglia are the resident immune cells patrolling the cerebral microenvironment. They account for around 10% of brain cells. They rapidly respond to local damage and undergo a change in morphology when activated [12]. In AD, many genetic factors are correlated with microglia. The most understood is Trem2. One of their physiological roles is to prune synapses in development and modulate plasticity. The reactivation of developmental pruning can be observed in the aged brain. Synapse loss is an important early event in AD pathogenesis and microglia has been implicated in synapse loss in AD [13]. Single-cell experiments have identified a subset of microglia called the disease-associated subset (DAM). According to this model, microglia have a mechanism that senses damage to neurons (NAMPs) via specific receptors like TREM2.

The typical AD patient has an amnestic phenotype that progresses to impairment in language, spatial cognition, executive functions and working memory. The amnestic presentation is more typical of late-onset AD. Non-amnestic presentations are clinically more common in early-onset disease. Neuropsychiatric symptoms often co-occur. Non-amnestic phenotypes include: posterior cortical atrophy (also known as visual variant), in which visuospatial deficits predominate; the logopenic phenotype, which is a primary progressive, non-fluent aphasia with repetition difficulties; and a dysexecutive phenotype [14]. The diagnosis of AD has evolved from being purely pathological, as it was considered at the time of Alois Alzheimer, to purely clinical to purely biological (thanks to advancements in biomarkers) [15]. Mild cognitive impairment (MCI) constitutes the earliest symptomatic stage of AD, later progressing to overt clinical dementia as cognitive neurological signs become apparent and increasingly affect daily functioning [15]. With only clinical parameters, the diagnosis of AD was previously restricted only to the stage of dementia. Biomarker research has shown that biomarkers are positive well before the clinical onset of disease, thus stressing the importance of an AD continuum ranging from pre-clinical AD to MCI to dementia [16]. AD genes can be divided into causal and risk genes. In the 1990s, genetic studies discovered mutations in APP, PSEN1, and PSEN2, three genes involved in the processing of the amyloid precursor protein (APP) [4,17]. Various animal models were created to replicate amyloid pathology. These include vertebrate models like non-human primate models and canine models that replicate spontaneous AD, rodents and zebrafish models that enable the use of genetic tools and drug screening, and invertebrate models like Drosophila, C. elegans and yeast [18]. Refs. [18,19] Early-onset familial AD represents only a small percentage of all AD cases, which are mainly sporadic. Genetic variants have been associated with sporadic AD; however, no single genetic association is fully deterministic. Genetic studies of sporadic AD have challenged the neuron-centric view of the disease, suggesting that AD arises from alterations in cell–cell interactions in specific tissues of the brain [19].

Ongoing research is focusing on cell-specific interventions for Alzheimer’s disease (AD) that could potentially transform its treatment. These cell-specific interventions aim to be more precise and effective, minimizing side effects compared to current AD treatments. Specific interventions that are under development include, but are not limited to, Aβ-targeted CAR T-cell therapy, tau-targeted gene silencing, and astrocyte-derived exosomes. Although these interventions are in the early stages of development, they hold promise for revolutionizing AD treatment and offer hope to those affected by the disease (reviewed in [20].

Unfortunately, current in vitro models are limited in modelling the complexity of AD pathogenesis [21], which, in turn, limits the possibility of identifying effective strategies of intervention. 

Bulk and single-cell transcriptomics have unveiled the intricate cellular diversity and dynamics within aging and degenerating brains, notably in postmortem Alzheimer’s disease (AD) samples. This approach has pinpointed disease-related gene networks and pathways across various cell types. Despite the wealth of insights from these studies, the number of overlapping differentially expressed genes (DEGs) is relatively low, largely due to methodological differences and challenges in obtaining undamaged cells from post-mortem brain tissue. To enhance the reliability of postmortem transcriptomic studies, it will be necessary to address biological variability, optimize cell-isolation methods, standardize cell clustering annotation, and incorporate spatial cell transcriptomics (reviewed in [22].

In this study, we aimed to characterize the AD expression signature by performing a meta-analysis of multiple human datasets, including different cell populations from various brain regions, and to compare cell-specific alterations in AD patients and in vitro models to highlight the appropriateness and the limitations of the currently available models in recapitulating AD pathology.

The meta-analysis showed consistent enrichment of the Rho GTPases signaling pathway among different cell populations and in the models. Rho GTPases are a family of proteins that play a crucial role in regulating cellular functions, such as cytoskeleton modulation, growth cone and dendritic spine formation, and axonal guidance [23]. In the nervous system, Rho GTPases are highly expressed, as they are involved in neuronal development and synaptic plasticity. Notably, Rho GTPases have been implicated in neurodegenerative processes, including AD. The deposition of extracellular plaques in AD brains leads to the activation of the Rho-GTPases pathway. This activation subsequently triggers the hyperactivation of GSK3beta, a kinase enzyme involved in tau hyperphosphorylation, tau aggregation, microtubule instability, and actin modulation [24]. These events are hallmark features of AD pathogenesis. 

Studying human cells using in vitro model systems is key to enhancing our understanding of AD mechanisms and the development of effective treatments. Future studies will need to recreate all major cellular components of the AD brain in a single in vitro model; for example, through 3D cell cultures, the incorporation of synthetic immune systems and the modelling of non-genetic factors such as sex and microbiome. We hope that our data will prove useful in indicating a starting point for the development of future, more reliable, in vitro models.

## 2. Materials and Methods

The NCBI transcriptomics database Gene Expression Omnibus (GEO) was searched in September 2022 to collect transcriptomic datasets of human and model AD brain samples. Three datasets generated from in vitro AD models were retrieved, each modelling a different cell type. GSE139643 for neurons, GSE187452 for microglia and GSE157461 for astrocytes (Table 1). In brief, GSE139643 included data from human neuroblastoma SH-SY5Y cells exposed to 5 μM Aβ-(1−42) for 24 h [25]; GSE187452 included data from human monocyte-derived microglial-like (MDMi) cells stimulated for 24 h to 500 nM stable human Aβ1-42 oligomers [26]); GSE157461 was generated using primary human astrocytes treated with 1 μM Aβ1−42 for 24 h [27]. Detailed experimental details can be retrieved from the indicated references.

For the human data, the human single-nucleus/single-cell RNA-seq datasets were obtained from The Alzheimer’s Cell Atlas (TACA) [28]. The GSE148822 and GSE157827 datasets were used for the present study. The GSE148822 included transcriptomes from post-mortem brain tissues of 10 AD donors and 8 controls. snRNA-seq was performed on 482.472 nuclei. For each donor, two brain cortical regions were analyzed: occipital cortex (OC) and occipitotemporal cortex (OCT). The GSE157827 included transcriptomes from post-mortem brain tissues of 12 AD donors and 9 controls. snRNA-seq was performed on 169.496 nuclei. For each donor, samples from prefrontal cortex (PFC) tissue were included. The characteristics of the included datasets are summarized in Table 2. 

The differential expression analysis of individual transcriptomic datasets was performed using GEO2R and GREIN. For the microarray dataset, the Linear Model of Microarray Analysis (LIMMA) was used for the identification of the Differentially Expressed Genes (DEGs). For the analysis of the RNA-seq, the negative binomial generalized linear model, as implemented in edgeR, was used. A False Discovery Rate (FDR) q-value < 0.005 was considered the threshold of statistical significance. The Fisher’s inverse Chi-square combined test was used for meta-analysis [29]. The following formula was used to combine *p*-values from the datasets:

$$\chi^{2}\left(df=2K\right)=-2\sum_{i=1}^{k}log(X_{i})$$

where $X_{i}$ represents the individual raw *p*-value for the *i*th gene and K represents the number of considered p-values. Overall *p*-values for each gene were then obtained from the $\chi^{2}$ values and compared to the reference value (*p* = 0.05) to determine statistical significance. We identified the list of overlapping genes between human data and the data from the in vitro model for each cell type and the statistical significance of the overlap for the up and down DEGs for each cell type was calculated using a modified Fisher exact Chi-square test. A *p*-value < 0.05 was considered statistically significant. 

**Figure 1:**
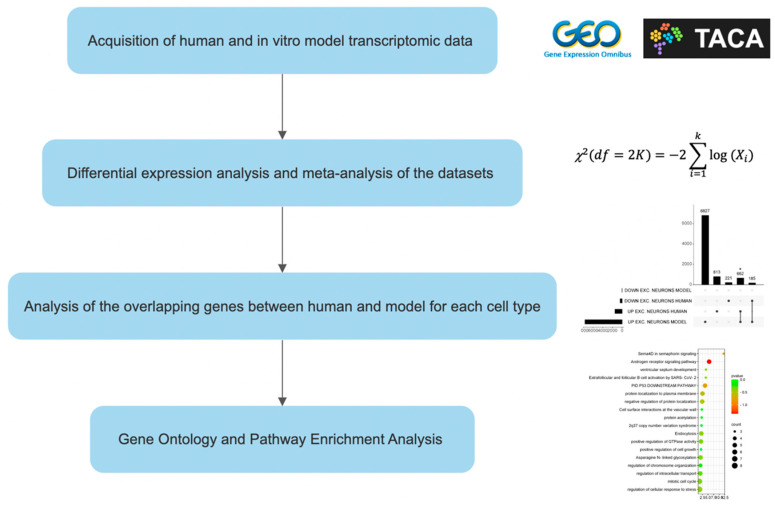
Experimental design. “*” means an extremely significant difference (*p* < 0.05).

Functional Enrichment Analysis of the identified overlap genes was performed using the online software Metascape (v3.5.20230501) [30]. Metascape analysis relies on several databases (Gene Ontology, KEGG, MSigDB, Reactome). Upregulated and downregulated genes were analyzed separately. The workflow of the study is presented as Figure 1.

## 3. Results

### 3.1. Meta-Analysis of Human Alzheimer’s Disease Transcriptomic Data 

**Figure 2:**
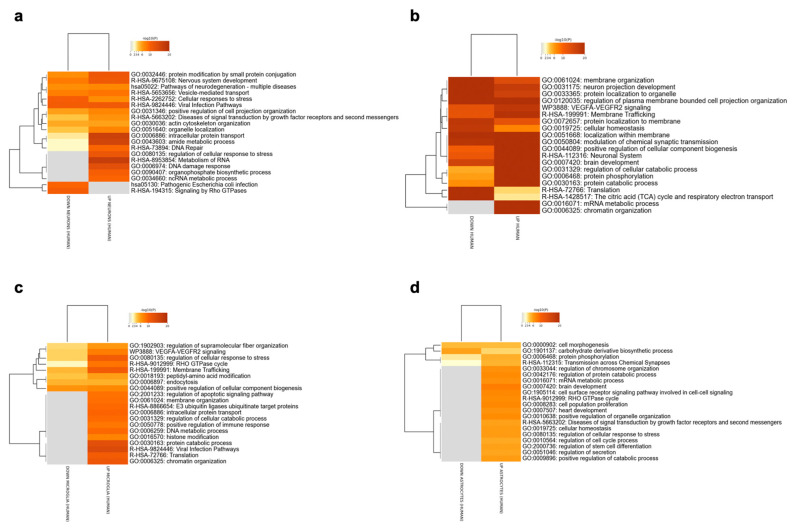
Enriched pathways among the DEGs identified for the excitatory neurons (**a**), inhibitory neurons (**b**), microglia (**c**) and astrocytes (**d**), using the meta-analysis of the RNA-Seq datasets GSE148822 and GSE157827.

The two human RNA-seq datasets GSE148822 and GSE157827, were meta-analyzed for the identification of the upregulated and downregulated DEGs for each brain cell population. The meta-analysis identified 1475 upregulated and 406 downregulated genes for neurons, 592 upregulated and 98 downregulated genes for astrocytes, and 854 upregulated and 186 downregulated genes for microglia. Enriched terms for excitatory neurons, astrocytes and microglia are shown in Figure 2. The Rho GTPase signaling pathway was enriched in all the meta-analyzed cell populations. Most of the enriched pathways were related to the functional role of Rho GTPases (cytoskeleton organization and modulation, cell projections’ regulation and intracellular transport) among all three cell populations. 

Although it was not possible to meta-analyze inhibitory neurons data due to the lack of multiple datasets, we included inhibitory neurons in the analysis for later comparison with the model. A total of 6602 upregulated and 8301 downregulated genes were identified for inhibitory neurons. Enrichment results are shown in Figure 2b. 

### 3.2. Relevance of an In Vitro Model of AD Neurons

**Figure 3:**
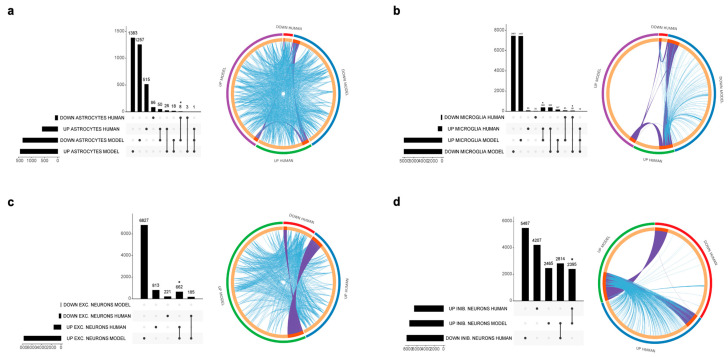
UpSet plots (**left**) showing the overlap of the DEGs identified from the AD patients and the in vitro models and circle plots (**right**), graphically showing overlapping genes and pathways. (**a**) Astrocytes, (**b**) microglia, (**c**) excitatory neurons, (**d**) inhibitory neurons. * *p* < 0.05.

**Figure 4:**
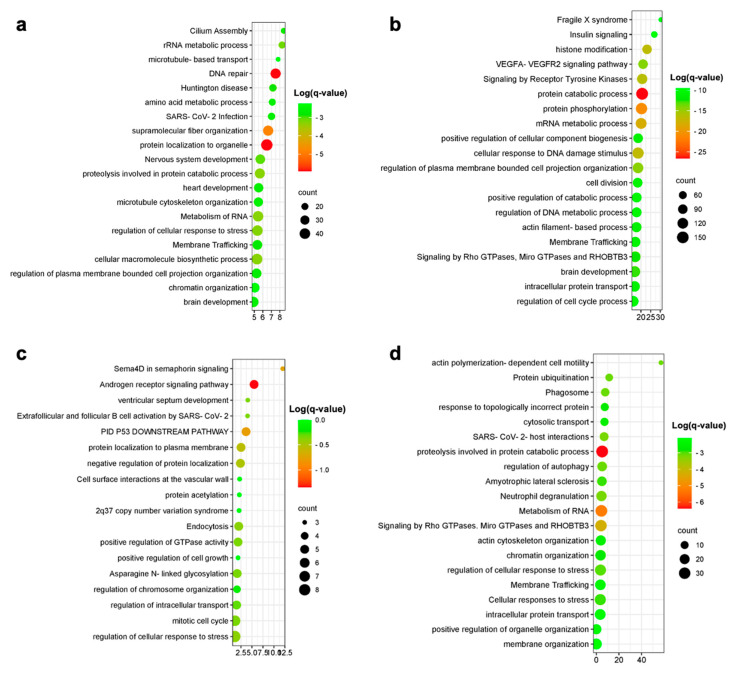
Enrichment analysis results for excitatory neurons (**a**), inhibitory neurons (**b**) upregulated overlap genes and microglia-upregulated (**c**) and -downregulated (**d**) overlap genes.

Excitatory and inhibitory neurons were separately analyzed. A total of 662 overlap genes were found between human and in vitro model data for the excitatory neurons, representing only 8.6% of the model genes. A significant overlap was observed for the upregulated DEGs (*p* < 0.05) (Figure 3c). The most statistically significant enriched terms obtained from the gene ontology and enrichment analysis are shown in Figure 4.

Among the upregulated DEGs characterizing the AD inhibitory neurons, we identified 2395 genes overlapping those from the in vitro model, representing 31.2% of all the in vitro model DEGs (*p* < 0.05) (Figure 3d). Enriched pathways for the overlapping upregulated genes in inhibitory neurons are shown in Figure 4. No overlap was observed between the human and the in vitro model downregulated DEGs for both the excitatory and inhibitory neurons. 

### 3.3. Relevance of an In Vitro Model of AD Astrocytes

Astrocytes showed a modest overlap between upregulated genes from human and in vitro model data that did not reach statistical significance (26 genes, representing 1.9% of model genes), as shown in Figure 3. Downregulated genes show a significant overlap of eight genes, representing 0.6% of the in vitro model genes (*p* < 0.05). 

### 3.4. Relevance of an In Vitro Model of AD Microglia

A total of 382 upregulated genes were found to be in common between the human and in vitro model data, representing 4.5% of the model genes. Downregulated genes showed an overlap of 68 genes, representing 0.8% of the in vitro model DEGs (Figure 3). Enriched pathways among upregulated and downregulated overlap genes are shown in Figure 4. The most enriched terms among all cell populations included pathways functionally related to Rho GTPase signaling (microtubule-based transport, cytoskeleton organization and cell projection organization), thus showing an overlap in the enriched pathway between human and model genes, despite the low percentage of overlapping genes, as graphically evident from the circle plots shown in Figure 3. The 20 most significantly upregulated and downregulated human DEGs overlapping with the DEGs from the in vitro AD models are presented in Table 3.

## 4. Discussion

Alzheimer’s disease (AD) is a devastating neurodegenerative disorder that predominantly affects the elderly, characterized by progressive cognitive decline, memory impairment, and functional loss. Despite extensive research efforts, the precise etiology and pathogenesis of AD remain elusive. Studying individual cell populations and their contributions to AD pathogenesis is crucial for unraveling the complex molecular mechanisms underlying the disease. In vitro models, which allow for the isolation and study of specific cell types, have emerged as indispensable tools in investigating neurodegenerative disorders [31]. In this study, we conducted a comprehensive meta-analysis of publicly available AD human transcriptomics datasets, and performed an enrichment analysis of the differentially expressed genes. Additionally, we explored the accuracy of in vitro models in recapitulating cell-specific pathogenic features of AD. Our findings shed light on the relevance and limitations of in vitro models and highlight the need for more sophisticated in vitro models to better represent the complexity of AD.

In vitro models have revolutionized biomedical research by providing controlled experimental systems to study biological processes and diseases. For neurodegenerative disorders like AD, in vitro models offer the advantage of studying individual cell populations in isolation, enabling researchers to investigate the specific role of neurons, microglia, astrocytes, and other cell types in disease pathogenesis. Furthermore, these models allow for the high-throughput screening of potential therapeutic agents and the evaluation of drug efficacy. However, it is important to recognize that in vitro models have inherent limitations as they lack the intricate interactions between different cell types, cellular microenvironments and external stimuli present in the complex in vivo setting.

To gain a comprehensive understanding of the molecular landscape underlying AD, we conducted a meta-analysis of publicly available AD human single-cell transcriptomics datasets. Specifically, we analyzed different cell populations, including excitatory and inhibitory neurons, microglia and astrocytes, to gain insights into their unique contributions to AD pathogenesis. 

Of note, some genes well-known to be expressed in neurons and microglia by the recent supporting evidence were not accurately recapitulated in the models. For example, TREM2 expression in microglia was shown to play an important role in disease pathogenesis, enabling these cells to sense neuronal damage [32]. TRIM11 was found to have an important role in removing protein tangles and to be downregulated in AD neurons [33]. P38 MAPK was found to regulate Aβ toxicity [34]. Both TRIM11 and p38 MAPK were not present among the significant overlapped genes, raising concerns for future studies testing their use as therapeutic targets.

Among the up-regulated genes identified in our meta-analysis, we observed a consistent enrichment of the Rho GTPases signaling pathway in excitatory human AD neurons, microglia, and astrocytes.

Rho GTPases, particularly ROCK2, emerge as key players in neurodegeneration. In AD, ROCK2 inhibition shows promise in preserving synapse structure and promoting the autophagic clearance of the pathological Tau protein.

Furthermore, Rho GTPases affect neurodegeneration through their role in regulating the actin cytoskeleton. Cofilin-actin rods, implicated in early-stage neurodegeneration, are formed during neuronal stress. In AD, cofilin dephosphorylation, triggered by Aβ, induces Cdc42 activation and RhoA down-regulation, contributing to rod formation. Studies in AD mouse models suggest that enhancing cofilin phosphorylation via the overexpression of LIMK1 improves memory formation, underscoring the importance of Rho GTPase downstream effectors in neurodegeneration. The identification of the Rho GTPases signaling pathway as being consistently enriched in multiple cell populations in our study highlights its potential as a therapeutic target for intervention. Several Rho GTPase inhibitors are currently available and were tested in preclinical research. Early studies focused on nonsteroidal anti-inflammatory drugs (NSAIDs), such as sulindac sulfide, ibuprofen and indomethacin, which primarily function as cyclooxygenase (COX) inhibitors, and also exert RhoA activity. In cell studies involving SH-SY5Y cells transfected with the Swedish mutant APP695, and in the AD transgenic PDAPP mouse model, NSAIDs demonstrated their ability to lower Aβ42 formation and inhibit RhoA activity. Another approach involved the use of Rho kinase (ROCK) inhibitors, such as Y27632. This compound, originally identified as an antihypertensive drug, has shown to be able to lower Aβ levels in 2-month-old PDAPP mice and exhibited potential therapeutic effects. Moreover, NSC23766, a Rac1 inhibitor, which operates by preventing the interaction between Rac1 and its guanine nucleotide exchange factors (GEFs), unveiled the capacity to decrease APP and Aβ levels through APP gene regulation, suggesting its potential role in regulating Aβ metabolism. Additionally, it demonstrated an ability to protect against Aβ42-peptide-induced cell death. Along the same lines, another Rac1 inhibitor, EHT1864, efficiently altered APP metabolism processing by selectively inhibiting γ-secretase metabolism. While RhoA and Rac1 have received substantial attention, studies targeting the third member of the Rho GTPase family, Cdc42, have been relatively limited. However, recent drug design and development efforts have resulted in the discovery of several Cdc42 inhibitors, opening up a new avenue for exploring the role of Cdc42 in AD pathogenesis. Overall, the identification of specific pharmacological tools targeting the Rho GTPase pathway offers a viable means to investigate its contribution to AD pathogenesis and novel potential therapeutic intervention (as reviewed in [35]).

To assess the relevance and accuracy of in vitro models in recapitulating AD pathogenesis, we compared the results of our meta-analysis with transcriptomics data obtained from in vitro models of AD for each cell population. We found a relatively low number of overlapping DEGs between human and in vitro model data, suggesting that the current in vitro models have limited fidelity in replicating the complexities of AD pathogenesis observed in human brain tissue. However, our analysis revealed that in vitro models showed a higher fidelity in modeling up-regulated genes in neurons compared to microglia and astrocytes. On the other hand, the modeling of down-regulated genes was particularly challenging across all cell populations. This suggests that current in vitro models may not fully capture the complexity of gene regulation in the context of AD, especially as regards the down-regulated pathways.

Our study highlights the pressing need for the development of more sophisticated in vitro models that can better replicate the complexities of AD pathogenesis observed in human brain tissue. Although in vitro models may not fully recapitulate the entire disease complexity, they remain valuable tools for studying individual cell populations and screening potential drugs. Efforts should be directed towards creating more advanced in vitro models, such as brain organoids and 3D cultures, which can better mimic the cellular interactions and environmental factors present in the brain microenvironment.

Additionally, integrating multiple omics data, including epigenetic and protein-level alterations, holds promise in providing a more comprehensive understanding of the relevance of in vitro models in AD research. Studying epigenetic changes, such as DNA methylation and histone modifications, can shed light on the dynamic regulation of gene expression in AD. Similarly, analyzing protein-level alterations can reveal post-transcriptional modifications that might not be evident in transcriptomics data alone.

Despite the valuable insights gained from our meta-analysis, our study suffers from several significant limitations, particularly the limited number of datasets available for the meta-analysis. The current scarcity of datasets, combined with the low representation of cell populations and brain regions in our analysis, poses constraints regarding the breadth and generalizability of our findings. This limitation highlights the necessity for future studies to adopt a more inclusive approach by incorporating a diverse range of datasets, accommodating a variety of cell types that play pivotal roles in AD pathogenesis. This comprises, but is not limited to, the inclusion of endothelial cells and pericytes, given their roles in both AD pathogenesis and the maintenance of blood–brain barrier integrity.

Expanding the dataset pool not only serves to increase the statistical robustness of our findings but also ensures a more comprehensive exploration of the molecular landscape of AD. Incorporating a broader range of brain regions, particularly those affected in AD, such as the hippocampus and entorhinal cortex, is also strongly warranted. These brain regions are known to undergo significant pathological changes in AD, and their inclusion in analyses can provide a more detailed understanding of the disease’s progression.

In light of these points, future efforts should be directed towards actively collecting and integrating additional datasets. This approach aims to enrich the diversity of our analyses, enabling a more representative and insightful exploration of the molecular alterations underlying AD. By addressing the current limitations in dataset availability, it is possible to enhance the reliability and applicability of our present findings, ultimately achieving a more thorough comprehension of the complex molecular dynamics characterizing AD pathogenesis.

Moreover, in the current study, we did not investigate the overlapping pattern of gene expression modulation that occurs in AD patients and in animal models of the disease. Future works will be devoted to analyzing how closely the cell-specific gene expression modulation of the different available animal models mirror the transcriptional alterations observed in AD patients.

Furthermore, our study focused solely on transcriptomics data, and an analysis of epigenetic components and protein-level alterations in AD in vitro models could enhance our understanding of disease mechanisms. Integrating multiple omics data may uncover novel regulatory networks and potential biomarkers that could be relevant to AD pathogenesis.

Another limitation of the present study comes from the use of SH-S5Y5 cells stimulated with Aβ for comparison with both excitatory and inhibitory AD neurons. Originating from neuroblastoma, SH-SY5Y cells carry unique characteristics associated with their tumoral phenotype and, despite efforts to induce differentiation, these cells fall short in achieving a maturity level akin to adult human neurons. Therefore, SH-SY5Y cells have limited reliability in resembling the diverse properties of excitatory and inhibitory neurons found in the brain. Furthermore, their simplistic 2D culture systems lack the nuanced three-dimensional structure crucial for replicating the complexity of the brain. In addition, being a single-cell type model, they overlook the intricate interplay of the various neuron types present in the brain, a crucial aspect of AD pathology.

To overcome the limitations of the current in vitro models, future research should focus on advancing the complexity and fidelity of these models. Organoids [36], three-dimensional (3D) cultures [37] and microfluidic devices represent promising avenues for the development of more sophisticated in vitro systems. Organoids are self-organizing, multicellular structures that recapitulate aspects of organ development and function, including the brain. They can be derived from patient-specific induced pluripotent stem cells (iPSCs) and allow for the study of complex cellular interactions and the three-dimensional architecture present in the brain. Incorporating different cell types within organoids, such as neurons, astrocytes, and microglia, could provide a more holistic representation of the AD brain microenvironment.

Similarly, 3D cultures enable the co-culture of different cell types in a three-dimensional setting, allowing for researchers to investigate cell–cell interactions and cell responses in a more physiologically relevant context. These models offer better insights into the cross-talk between different cell populations and how their interactions influence disease progression. Microfluidic devices, on the other hand, provide precise control over the microenvironment surrounding the cells and can simulate the blood–brain barrier, enabling the study of cell–cell interactions across this critical interface.

Moreover, the incorporation of patient-derived iPSCs into in vitro models holds great potential for personalized medicine approaches in AD research. Patient-specific iPSCs can be differentiated into various cell types affected by AD, providing a unique opportunity to study individualized disease mechanisms and identify patient-specific therapeutic targets.

In parallel, exploring the use of co-culture systems that mimic the complex cellular interactions within the brain microenvironment is crucial. AD pathogenesis involves a delicate interplay between neurons, astrocytes, microglia, and other cell types. Understanding how these interactions influence disease progression can open new avenues for drug development and precision medicine in AD.

## 5. Conclusions

In conclusion, our meta-analysis of AD human transcriptomics data and investigation of in vitro models shed light on the complexity of AD pathogenesis and the relevance of current in vitro systems. The Rho GTPases signaling pathway emerged as a key player in AD pathogenesis across multiple cell populations, warranting further investigation using in vitro models and potential therapeutic targeting. While in vitro models provide valuable insights into individual cell populations and drug screenings, their current limitations, particularly in modeling down-regulated genes and complex cellular interactions, emphasize the need for more sophisticated models.

The future of AD research lies in the development of advanced in vitro models, such as organoids, 3D cultures, and co-culture systems, which can better mimic the complexity of the brain microenvironment. Additionally, the integration of multiple omics data, including transcriptomics, epigenomics, and proteomics, could enhance our understanding of AD pathogenesis and identify novel therapeutic strategies.

By combining insights from meta-analyses of human transcriptomics data and cutting-edge in vitro models, we can work towards unraveling the intricacies of AD pathogenesis and eventually pave the way for effective therapeutic interventions to combat this devastating neurodegenerative disorder.

## Figures and Tables

**Table 1:** Datasets used in the study for the in vitro models.

GEO Accession	Model Description
GSE139643	Abeta-stimulated neuroblastoma SH-SY5Y cells
GSE187452	Abeta-stimulated human monocyte-derived microglia cells
GSE157461	Abeta-stimulated primary human astrocytes

**Table 2:** Datasets used in the study for the human AD.

GEO Accession	Brain Region	N. of Samples
GSE148822	Occipital cortex (OC) and occipitotemporal cortex (OCT)	10 AD, 8 CTR
GSE157827	Prefrontal cortex (PFC)	12 AD, 9 CTR

**Table 3:** The 20 most significantly upregulated and downregulated human DEGs overlapping with the DEGs from the in vitro AD models.

UP EXC. NEURONS	UP INIB. NEURONS	UP MICROGLIA	DOWN MICROGLIA	UP ASTROCYTES	DOWN ASTROCYTES
RNF180	CLDN1	SSBP1	C7orf50	GABBR1	OSBPL9
PIK3IP1	RNF180	ARHGEF37	PRPSAP2	SDC4	ZNF91
FOXP1	SLC8A1-AS1	TRIM25	SP140	BTRC	ZNF442
SREK1	COL12A1	SLC8A1-AS1	ARID3A	GTF2H2	AUTS2
MSC-AS1	ZNF154	FRA10AC1	MAGT1	ZNF654	PCCA
MAPK1IP1L	C11orf1	SLC7A6OS	SUFU	GBA	TRIM2
RERG	PIK3IP1	ARL17A	DHTKD1	BCAP29	PHIP
SESN3	ARNT	FAM13A	PID1	TMED9	ARSG
TMEM232	PIAS2	OPRM1	FOXO1	STOML1	
ZC3H15	MEGF10	TK2	ARID1B	INTS2	
CD109	GNA13	LINC02328	PFKFB4	SLC35F5	
SYNJ1	TRPC4	DLST	GRIPAP1	RPAP2	
PDXDC1	CCNL1	MICU3	MAPKAPK3	KDSR	
GRM8	SAV1	DNAH14	NEK6	TRIM44	
SLC48A1	NT5E	DIAPH2-AS1	RAB40C	RPP30	
DENND6B	MTMR7	EFHC1	ARHGAP35	REX1BD	
CFAP221	TNXB	RNF217	RCAN1	CHST7	
EMCN	SEC14L2	AUTS2	SEMA4D	PIAS1	
PRKAA2	PTPRD	G2E3	PGPEP1	DENR	
SERP2	SREK1	ZNF718	ROCK2	DYM	

## Data Availability

All data used for the present study can be downloaded from the Gene Expression Omnibus GEO; https://www.ncbi.nlm.nih.gov/gds, (accessed on 2 May 2023) database, under accession numbers GSE139643, GSE187452, GSE157461, GSE148822 and GSE157827.
